# 

**Published:** 1901-06-01

**Authors:** 


### NOTICE TO CORRESPONDENTS.

All MSS., Letters, Books for Review, and other matters intended for the Editor should be addressed The Editor, "The Hospital," The Hospital Building, 28 & 29 Southampton Street, Strand, W.C.

The Editor cannot undertake to return rejected MS., even when accompanied by stamped directed envelope.

### Notice to Advertisers and Subscribers.

All Advertisements, Orders for Copies of The Hospital, and Business Communications should be addressed to **THE MANAGER (Not to the Editor),** The Hospital, 28 & 29 Southampton Street, Strand, London, W.C.

The Cost of Subscription, post free, is as follows:—Per week, 2½d.; per quarter, 2s. 8d.; per half-year, 5s. 3d.; per annum, 10s. 6d. Foreign:—Per annum, 15s. 2d., payable, in all cases, in advance.

**NOTICE.—Vol. XXIX. of "The Hospital" (October 1900, to March, 1901), in handsome cloth binding are now on sale at the Office of this Journal, price, 7s. 6d. Cases for binding the Numbers contained in this Volume 1s. 6d. Index to Vol. XXIX., 2½d., post free.**

**The Monthly Part for June is now ready. Price 6d., by post 8d.**

### SCRAPS AND GLEANINGS.

THE new hospital which has been erected at Newport, Mon., is to be formally opened by Lord Tredegar on Bank Holiday next, August 5th. The buildings contain accommodation for 84 beds, and it is expected they will cost upwards of £30,000, exclusive of the cost of furnishing.

THE site on which the new joint hospital at Havock, Dumbarton, is built consists of five acres, on which are built six different buildings. The hospital has taken three years to build, and was estimated to cost about £14,000. It has now been formally opened by Lord Overtoun.

THE special report on the open-air treatment of pulmonary tuberculosis, which was presented at the annual meeting of the National Sanatorium for Consumption, Bournemouth, showed that a large percentage of the cases of arrested disease were in the early stage, and only 11 per cent. were in the advanced stage.

THE Executive Committee on the reconstruction of the Glasgow Royal Infirmary have drawn up a lengthy report which states that after full consideration of all the circumstances the committee are convinced that the only effectual plan will be to entirely reconstruct the whole of the Infirmary. This will cost at least £250,000, of which sum the committee have upwards of £80,000 in hand.

### THE STUDENT OF MEDICINE.

### APPOINTMENTS.

**H. M. Brownfield**, L.R.C.P.Edin., M.R.C.S.Eng., appointed Certifying Factory Surgeon for the Petersfield Division of Hampshire. **E. A. Chartres**, F.R.C.S. and L.R.C.S.I., appointed Senior Medical Officer Northern Nigerian Protectorate Colonial Government. **J. B. Dunlop**, B.A.Cantab., M.R.C.S., L.R.C.P., appointed Resident Medical Officer to the Swansea Hospital. **Frederick Hudson Evans**, M.R.C.S.Eng., L.R.C.P.Lond., appointed Medical Superintendent at the Mendip Hills Sanatorium for Consumption, Hill Grove, Wells, Somerset. **A. E. Harrisson**, M.B., B.C.Cantab., appointed Certifying Factory Surgeon for the Daventry District of Northants. **Arthur George Haydon**, M.D., M.R.C.S., appointed Medical Officer in charge of the Suffolk Regiment; and Civil Surgeon, Royal Army Medical Corps. **R. G. Hogarth**, F.R.C.S.Eng., appointed Assistant Surgeon to the General Hospital, Nottingham. **William Hunter**, M.D.Edin., F.R.C.P.Lond., appointed Examiner in Medicine (Systematic and Clinical) in the University of Glasgow. **Cuthbert Lockyer**, M.D., B.S., M.R.C.P.Lond., F.R.C.S.Eng., appointed Obstetric Registrar to Charing Cross Hospital. **Reginald S. Nichol**, M.B., Ch.B.Vict. Univ., appointed Senior Resident Medical Officer to St. Mary's Hospital, Manchester. **C. H. R. Pentreath**, M.B., B.C., B.A.Camb., appointed Honorary Physician to the Auckland Hospital, New Zealand. **F. W. Pollard**, B.A., Lic. Med. Surg. and Mid. Univ. Dub., appointed Medical Officer and Public Vaccinator for the No. 2 District of the Blackburn Union. **W. Sykes**, M.D., M.R.C.S., L.R.C.P., appointed Honorary Physician to the St. Peter's Convalescent Home, Maybury, Woking. **F. E. Taylor**, M.A., M.Sc., M.B., Ch.B., appointed Resident Medical Officer at the Chelsea Hospital for Women. **A. Thompson**, M.R.C.S., L.R.C.P., appointed Medical Officer to the Newbury Union Workhouse. **A. C. K. Turner**, M.B., C.M.Edin., appointed Certifying Factory Surgeon for the Fairford District of Gloucestershire. **H. Wilcox**, M.B.-Aberd., M.R.C.S.Eng., appointed Certifying Factory Surgeon for the Fleet District of Hampshire.

ST. THOMAS'S HOSPITAL MEDICAL AND SURGICAL COLLEGE.—House Appointments.—House Physicians: **Z. Mennell**, L.R.C.P., M.R.C.S.; **F. B. Skerrett**, B.Sc.Lond., L.R.C.P., M.R.C.S.; **J. E. H. Sawyer**, M.A., M.B., B.Ch.-Oxon; **J. L. Lock**, B.A., M.B., B.C.Cantab., L.R.C.P., M.R.C.S. Assistant House Physicians: **L. H. C. Birkbeck**, B.A., M.B., B.Ch.Oxon; **V. S. Hodson**, B.A., M.B., B.Ch.-Oxon. House Surgeons: **T. H. Edwards**, L.R.C.P., M.R.C.S.; **B. S. Wills**, L.R.C.P., M.R.C.S.; **T. S. Taylor**, M.A., M.B., B.C.Cantab.; **T. Burfield**, M.A., M.B., B.C.Cantab., L.R.C.P., M.R.C.S. Assistant House Surgeons: **C. A. R. Nitch**, M.B.Lond., L.R.C.P., M.R.C.S.; **W. H. O. Woods**, B.A., M.B., B.C.Cantab.; **S. Hunt**, L.R.C.P., M.R.C.S. **A. S. Grimwade**, L.R.C.P., M.R.C.S. Obstetric House Physicians: (*Senior*) **A. E. Martin**, M.A., M.B., B.C.Cantab., L.R.C.P., M.R.C.S.; (*Junior*) **H. S. Stannus**, L.R.C.P., M.R.C.S. Clinical Assistants: (*Throat*) **H. M. Harwood**, M.A., M.B., B.C.Cantab.; **A. E. Stevens**, M.B.Durh., L.R.C.P., M.R.C.S.; (*Skin*) **W. C. Mence**, L.R.C.P., M.R.C.S.; **T. D. Miller**, L.R.C.P., M.R.C.S.; (*Ear*) **H. M. Harwood**, M.A., M.B., B.C.Cantab.; (*Electrical Department*) **L. S. Dudgeon**, L.R.C.P., M.R.C.S.; (*X Ray Department*) **R. Small**, L.R.C.P., M.R.C.S.

28 & 29 SOUTHAMPTON STREET, STRAND, W.C.

